# Risk Assessment of Smokeless Tobacco among Oral Precancer and Cancer Patients in Eastern Developmental Region of Nepal

**DOI:** 10.31557/APJCP.2019.20.2.411

**Published:** 2019

**Authors:** Jyotsna Rimal, Ashish Shrestha, Iccha Kumar Maharjan, Sushmita Shrestha, Priyanka Shah

**Affiliations:** 1 *Department of Oral Medicine and Radiology,*; 2 *Department of Public Health Dentistry, *; 3 *Department of Conservative Dentistry and Endodontics B.P. Koirala Institute of Health Sciences, Dharan,*; 4 *Department of Pedodontics, Nepal Medical College, Kathmandu, Nepal. *

**Keywords:** Epidemiology, oral potentially malignant disorders, oral cancer, risk assessment, smokeless tobacco

## Abstract

**Background:**

Oral potentially malignant disorders (OPMDs) and oral cancer (OC) are preventable oral mucosal diseases prevalent in Asian region. This epidemiological study aims to identify oral potentially malignant disorders (OPMDs) and oral cancer (OC), confirm histopathologically, and treat or refer these cases among the population of Eastern Development Region (EDR) of Nepal. It also attempts to assess the risk factors associated in order to compare dose–response measurements of oral habits in these patients.

**Methods:**

Cross-sectional epidemiological study was conducted over a period of 2 years in 16 districts of EDR. A total of 3,200 people were screened. A brief history was taken and visual screening examination was conducted in two phases as described by British Columbia Oral Cancer Prevention Program. Suspicious oral lesions were biopsied either by punch or scalpel after toluidine blue staining. Tissue specimen was transported to the institutional lab for histopathological processing. The reports were sent to the patients through the local leaders or organizations.

**Results:**

More than 40% of the study population either chewed areca nut and/or tobacco. Eighteen percent were smokers. OPMDs were prevalent among 468 study population with male-female ratio of 3:1. Tobacco pouch keratosis (50.4%) was the most prevalent OPMD, followed by OSF (29.1%). Fifty-two had squamous cell carcinoma and 8 had verrucous carcinoma.

**Conclusion:**

Chewing areca nut, tobacco, commercial areca nut/tobacco preparation and smoking being the major risk factors, there is high prevalence of oral cancer and OPMDs in the EDR of Nepal.

## Introduction

Oral potentially malignant disorders (OPMDs) and oral cancer (OC) are associated with betel quid chewing (Rimal and Shrestha, 2015). Betel quid chewing comprises of areca nut, slaked lime, betel leaf with or without tobacco. Arecanut is the fourth most addictive substance globally and is socially acceptable habit in South Asia (Gupta and Warnakulasuriya, 2002; Balasimha and Rajagopal, 2004). Arecanut is often chewed in a form of betel quid. Generally, a betel quid comprises of arecanut, slaked lime, betel leaf with or without tobacco. Nowadays, these quids are available commercially in sachets and are popular in public due to vigorous marketing strategy of these products (Gupta and Warnakulasuriya, 2002). The incidence of OPMDs is becoming an epidemic, targeting the younger generation. These habits have been associated with lesions like leukoplakia, erythroplakia, oral submucous fibrosis (OSF), chewer’s mucosa, periodontal diseases etc. ultimately may lead to OC. Many of these are preventable and curable if diagnosed early (Kerr et al., 2011). Most of the OPMDs are diagnosed on routine oral examination (Rimal and Shrestha, 2015). In Nepal, there is no epidemiological data on the prevalence of oral mucosal lesions with focus on oral precancer or cancer. Epidemiological studies have shown the relationship between OC risks and tobacco chewing among men (Madani et al., 2010; Zini et al., 2010; Yang et al., 2010). The aim of this study was to identify OPMDs and oral cancer, confirm histopathologically, treat or refer patients and also to assess the risk factors associated with oral habits.

## Materials and Methods

A cross-sectional epidemiological study was carried out from October 2012 to October 2014. Approval from Institutional ethical review board (IERB) (Acad. 478/069/070) of B.P. Koirala Institute of Health Sciences, along with ethical approval from Nepal Health Research Council (Reg. no. 128/2012) were obtained. Pamphlets relating to oral health, tobacco use, OPMDs and OC were designed and printed for distribution. The screening camps were organized with the assistance of local clubs/organizations/leaders like Rotary clubs and/or youth club in each district. The public were informed well ahead of time of camp through FM radio for dissemination of the information of the screening camps.

**Table 1:** Association of Demographic Characteristics, Alcohol Habits, Oral Hygiene and Periodontal Health with OC and OPMDs (n = 3,200)

Variables/ Categories	Frequencyn (%)	OR^c^	95% CI
Age in years			
<30	684 (21.4)	1	-
30-45	1,844 (57.6)	3.48	2.51 – 4.82
>45	672 (21.0)	3.42	2.38 – 4.91
Sex			
Female	1,600 (50.0)	1	-
Male	1,600 (50.0)	0.3	0.24 – 0.37
Alcohol			
No	2,872 (89.8)	1	-
Yes	328 (10.2)	4.21	3.29 – 5.37
Alcohol duration			
No	2,872 (89.8)	1	-
≤20 years	196 (6.1)	5.53	4.09 – 7.46
>20 years	132 (4.1)	2.71	1.84 – 3.99
Periodontal pockets			
No	2,588 (80.9)	1	-
Yes	612 (19.1)	3.96	3.23 – 4.87
Gingival recession			
No	2,212 (69.1)	1	-
Yes	988 (30.9)	9.72	7.83 – 12.06
Oral hygiene			
Good	464 (14.5)	1	-
Fair	1,684 (52.6)	2.85	2.01 – 4.47
Poor	1,052 (32.9)	7.5	3.12 – 9.47

Statistical significance, p<0.20; OR: Crude odds ratio; 95% CI: 95% confidence interval

**Table 2:** Association of Arecanut, Smokeless Tobacco and Smoking Habits with OC and OPMDs (n = 3,200)

Variables/ Categories	Frequency n (%)	OR^c^	95% CI
Chewing habit			
No	1,884 (58.8)	1	-
Arecanut	772 (24.2)	5.23	3.09 – 8.28
Tobacco	544 (17.0)	6.24	4.67 – 10.64
Arecanut			
No	2,472 (77.2)	1	-
Yes	728 (32.8)	2.47	2.02 – 3.02
Arecanut + betel leaf			
No	2,944 (92.0)	1	-
Yes	256 (8.0)	6.36	4.87 – 8.30
Arecanut +betel leaf +lime			
No	2,976 (93.0)	1	-
Yes	224 (7.0)	7.36	5.47 – 9.63
Arecanut + betel leaf + lime + catechu			
No	2,996 (93.6)	1	-
Yes	204 (6.4)	5.27	3.93 – 7.07
Arecanut + betel leaf + lime + catechu + tobacco			
No	3,024 (94.5)	1	-
Yes	176 (5.5)	10.97	7.93 – 15.17
Commercial tobacco preparation			
No	2,780 (86.8)	1	-
Yes	420 (13.2)	10.92	8.70 – 13.72
Tobacco			
No	2,412 (75.3)	1	-
Yes	788 (24.7)	3.73	2.31 – 4.64
Chewing habit duration			
No	1,884 (58.8)	1	-
≤20 years	180 (5.7)	2.75	1.65 – 4.62
>20 years	1,136 (35.5)	4.89	1.28 – 6.89
Chewing time			
No	1,884 (58.8)	1	-
≤20 minutes	1,048 (32.8)	2.06	1.39 – 2.81
>20 minutes	268 (8.4)	5.17	3.02 – 7.72
Chewing frequency			
No	1,884 (58.8)	1	-
≤10 times/day	1,144 (35.8)	2.73	1.18 – 2.30
>10 times/day	172 (5.4)	3.08	1.16 – 6.42
Smoking			
No	2,796 (87.4)	1	-
Yes	404 (12.6)	2.77	2.19 – 3.51
Smoking frequency			
No	2,796 (87.4)	1	-
≤10 times/day	328 (10.2)	2.64	2.04 – 3.42
>10 times/day	76 (2.4)	6.04	3.54 – 10.30
Smoking duration			
No	2,796 (87.4)	1	-
≤20 years	282 (8.8)	2.67	2.02 – 3.53
>20 years	122 (3.8)	3.87	2.61 – 5.74

Statistical significance, p<0.20; OR: Crude odds ratio; 95% CI: 95% confidence interval

**Table 3:** Multivariate Logistic Regression Model of Risk Factors for OC and OPMDs

Variables/ Categories	OR^a^	95% CI
Age in years		
<30	1	-
30-45	3.66	1.59 – 8.46
>45	3.5	2.18 – 5.63
Sex		
Female	1	-
Male	2.24	1.51 – 3.32
Chewing habit		
No	1	-
Arecanut	0.05	0.02 – 0.13
Tobacco	5.77	3.44 – 9.69
Arecanut		
No	1	-
Yes	3.75	2.45 – 5.98
Commercial tobacco preparation		
No	1	-
Yes	0.26	0.16 – 0.42
Chewing frequency		
No	1	-
≤10 times/day	0.24	0.15 – 0.40
>10 times/day	0.31	0.15 – 0.65
Smoking		
No	1	-
Yes	0.14	0.05 – 0.39
Smoking frequency		
No	1	-
≤10 times/day	0.26	0.10 – 0.71
>10 times/day	0.69	0.18 – 0.97
Smoking duration		
No	1	-
≤20 years	0.15	0.06 – 0.40
>20 years	0.42	0.08 – 0.84
Oral hygiene		
Good	1	-
Fair	3.16	2.12 – 4.38
Poor	8.07	3.51 – 13.16

Statistical significance, p<0.05; OR: Adjusted odds ratio; 95% CI, 95% confidence interval

A total of 3,200 people, consisting of 200 from each district (using stratified cluster sampling) with age range of 16-70 years were screened for OPMDs and OC at the screening camps organized in 3 zones covering 16 districts of Eastern Developmental Region (EDR). Study site was designed as per WHO Oral Health Survey–Basic Methods. The study base was B.P. Koirala Institute of Health Sciences, Dharan. 

Scheduling for the main study was planned on the basis of pilot study conducted on randomly selected 50 individuals of Danda Bazaar, Dhankuta during an oral health camp. The psychometric properties of the questionnaire in terms of concurrent validity as well as internal and test-retest reliability were good. 

The self-administered questionnaire was used for demographic data and information regarding tobacco habits. A brief history was taken about general health history, oral hygiene habits, oral deleterious habits and symptoms of oral pain or discomfort in a pro forma. Visual screening examination was conducted as described by British Columbia Oral Cancer Prevention Program (Williams et al., 2008). Optional screening adjunct (Toluidine blue staining) was used for determining the site before the biopsy. Tissue specimen were transported to the institute’s laboratory for histopathological processing. On completion of data collection, knowledge regarding oral carcinogens and their effects on the oral cavity was provided to the study population. Oral health education was provided regarding maintenance of oral hygiene and oral urgent treatments. 

The histopathological reports along with referrals were sent via emails, to the local club/organization/leader for distribution to the patients. Individuals with OPMDs or OC were referred to BP Koirala Institute of Health Sciences Hospital for further management. 

The data was entered in SPSS 11.5 version and analyzed by logistic analysis to determine the association between habits and lesions. Odds ratios were used to associate different habits, their frequency and duration of use with the occurrence of OC and/or OPMDs. P values of less than 0.05 were considered to be statistically significant.

## Results

A total of 3200 participants, with equal gender distribution, were studied. The mean age of the population was 38.46 ± 14.42 with almost equal age group distribution. With regard to literacy of the population 84% could read and 88.3% could write. More than 40% of the study population either chewed areca nut and/or tobacco. Eighteen percent were smokers and less than 10% consumed some form of alcohol. Oral hygiene status of the population showed that more than 95% had stains and deposit in their teeth, almost 20% had periodontitis and nearly 30% suffered from receding gums. OPMDs were prevalent among 468 of the study population with male-female ratio of 3:1 and concentrated more in the plains. OPMDs diagnosed were tobacco pouch keratosis (TPK), leukoplakia, lichen planus, oral candidiasis and OSF. Area wise distribution showed that OPMDs were more prevalent in Morang district followed by Sunsari and Siraha. However, prevalence was also seen in hill districts namely, Taplejung, Ilam and Okhaldhunga. Solukhumbu district also showed notable number of OPMDs. Tobacco pouch keratosis (50.4%) was the most prevalent form of OPMD followed by OSF (29.1%). Labial vestibule, followed by floor of the mouth, were the most affected sites. Only 16 cases did not have chewing habits. Three hundred seventy-six smoked and 352 consumed alcohol.

Amongst the 3200-people surveyed, 60 of them had OC (40 males and 20 females) of which most were either in the range of 45-54 years or more than 64 years. Most of them belonged to the districts from the plains, namely Morang, Sunsari, Saptari and Jhapa. Only 4 patients of oral cancer did not chew areca nut and/or tobacco. Thirty-six of them were smokers and 16 of them consumed alcohol. Squamous cell carcinoma was seen among 52 patients and remaining had verrucous carcinoma. Buccal mucosa/vestibule was the most affected site followed by floor of the mouth. On Simplified Oral Hygiene Index (OHIS), 52.6% had fair oral hygiene and one third of the people had poor oral hygiene.

In order to assess the risk factors for OC and OPMDs, logistic analysis was carried out. The analysis shows association of OC and OPMDs with demographic characteristics, alcohol habits, and oral health conditions (Table 1). Logistic analyses were carried out for those parameters which were selected after sensitivity analysis. Odds ratio was >1 for variables like chewing areca nut, tobacco, commercial preparation and smoking (Table 2). Multivariate logistic analyses (Table 3) showed that disease risk increased with increase in frequency and duration of substance use and also with length of chewing of substance. Odds of disease occurrence increased with poor oral hygiene. 

## Discussion

Most of the people in the study were Hindu by religion and were farmer (semi-skilled) by profession. These data are consistent with the National Population and Housing Survey 2011 (Government of Nepal, 2011) and a study by Shrestha (2013). Almost 80% were married and more than 80% could read and/or write. The literacy rates differed from the National Report where the literacy rate of EDR is 67.1% (Government of Nepal, 2012). This difference in the data could be because the question asked in our study questionnaire was “Are you able to read? Are you able to write?” and had no question about the education of an individual. 

More than 40% of the population chewed either areca nut and/or tobacco. Eighteen percent were smokers and less than 10% consumed some form of alcohol. National sample survey on tobacco use (Government of Nepal, 2011) showed prevalence rate of overall smoking and tobacco use as 38.4% and 44.7%, respectively. In our study, chewing tobacco was almost double of smoking. Similar result was found in a Global Adult Tobacco Survey (GATS) 2009-2010 in India where ratio of smokeless to smoking tobacco was 2:1 (International Institute for Population Sciences, 2010). The findings regarding presence of stains, periodontitis and OHI-S imply poor oral health awareness among the population surveyed.

A study (Oakley et al., 2005) done on high school children in Micronesia detected oral leukoplakia in 13% and OSF in 8.8% followed by chewers mucosa and lichenoid reaction. In another hospital based study by Mathew et al., (2008) on 1190 patients, showed prevalence of OSF (2.1%), smokers palate 2.77%, betel and chewers mucosa 0.84% respectively. The variation in incidence of different OPMDs can be attributed to geographic location, lifestyle, frequency and duration of use of tobacco and alcohol products as well as the site of placement of smokeless tobacco products.

Gupta et al., (2014) reported an increased incidence of oral squamous cell carcinoma and verrucous carcinomas in patients chewing tobacco. Sawyer and Wood (1992) also reported an increased risk with increase in the frequency and duration of the habit. Though the nicotine content of chewing tobacco is much lesser than that in the smoking form, it is said to have more carcinogenic potential because it remains in contact with the oral mucosa for longer time. Tobacco-specific N-nitrosamines is present in higher concentrations in smokeless tobacco and the absorption is further enhanced in alkaline environments. These factors are known to influence the formation and levels of toxic chemicals in tobacco chewers and smokers which play a key role in the malignant transformation in the oral cavity. Among other factors which could modify the risk of oral lesions, alcohol appeared to increase the risk of OSF, leukoplakia, and lichen planus but not OC. Our findings are consistent with the finding of these studies (Gupta et al, 2014; Sawyer and Wood, 1992).

Our study involves population of EDR only hence, cannot be generalized to remaining part of the country. Further study with larger sample size including the nationwide data would provide true prevalence of these diseases in the country. 

There is high prevalence of OC and/or OPMDs in the study population. Chewing areca nut, tobacco, commercial areca nut/tobacco preparation and smoking are the major risk factors for OPMDs and OC. Areca nut chewing with or without tobacco consumption emerged as the strongest risk factor for OPMDs and OC. Oral hygiene was also strongly associated with these oral diseases. The high prevalence of TPK and OSF in substance abusers compared with the general population emphasized the need for oral examination on a routine basis. Similar study is needed to document such findings of population in other parts of the country. Our research is a milestone in the epidemiology of OPMDs and OC, which would pave a path to plan the Oral Health Policy and implement oral health programs focused in the community of the target districts.

## Funding Statement

The research was funded by University Grant Commission, Nepal under faculty research grant 068/069. 
